# Pulmonary cavitation: an under-recognized late complication of severe COVID-19 lung disease

**DOI:** 10.1186/s12890-020-01379-1

**Published:** 2021-01-12

**Authors:** Zaid Zoumot, Maria-Fernanda Bonilla, Ali S. Wahla, Irfan Shafiq, Mateen Uzbeck, Rania M. El-Lababidi, Fadi Hamed, Mohamed Abuzakouk, Mahmoud ElKaissi

**Affiliations:** 1 Department of Pulmonology, Respiratory Institute, Cleveland Clinic Abu Dhabi, Abu Dhabi, United Arab Emirates; 2 Department of Infectious Diseases, Medical Specialties Institute, Cleveland Clinic Abu Dhabi, Abu Dhabi, United Arab Emirates; 3 Department of Pharmacy Services, Cleveland Clinic Abu Dhabi, Abu Dhabi, United Arab Emirates; 4 Critical Care Institute, Cleveland Clinic Abu Dhabi, Abu Dhabi, United Arab Emirates; 5 Department of Allergy and Immunology, Respiratory Institute, Cleveland Clinic Abu Dhabi, Abu Dhabi, United Arab Emirates; 6 Imaging Institute, Cleveland Clinic Abu Dhabi, Abu Dhabi, United Arab Emirates

**Keywords:** COVID-19, SARS-CoV-2, Cavity, Pulmonary cavitation

## Abstract

**Background:**

Pulmonary radiological findings of the novel coronavirus disease 2019 (COVID-19) have been well documented and range from scattered ground-glass infiltrates in milder cases to confluent ground-glass change, dense consolidation, and crazy paving in the critically ill. However, lung cavitation has not been commonly described in these patients. The objective of this study was to assess the incidence of pulmonary cavitation in patients with COVID-19 and describe its characteristics and evolution.

**Methods:**

We conducted a retrospective review of all patients admitted to our institution with COVID-19 and reviewed electronic medical records and imaging to identify patients who developed pulmonary cavitation.

**Results:**

Twelve out of 689 (1.7%) patients admitted to our institution with COVID-19 developed pulmonary cavitation, comprising 3.3% (n = 12/359) of patients who developed COVID-19 pneumonia, and 11% (n = 12/110) of those admitted to the intensive care unit. We describe the imaging characteristics of the cavitation and present the clinical, pharmacological, laboratory, and microbiological parameters for these patients. In this cohort six patients have died, and six discharged home.

**Conclusion:**

Cavitary lung disease in patients with severe COVID-19 disease is not uncommon, and is associated with a high level of morbidity and mortality.

## Background

The novel coronavirus disease 2019 (COVID-19) pandemic has caused over 700,000 recorded deaths worldwide thus far [1]. Infection with the novel severe acute respiratory syndrome corona virus 2 (SARS-CoV-2) causes COVID-19 which can lead to pneumonia and severe acute respiratory syndrome. The typical abnormalities seen on computerized tomography (CT) of the chest in patients with COVID-19 lung disease have been well described [2–5], with a comprehensive review and meta-analysis [6] of 55 studies finding peripheral ground glass opacities in most, consolidation in 44% (95% CI 1–71%), air bronchograms in 43% (95% CI 8–80%), linear opacities in 41% (95% CI 7–65%), crazy-paving pattern in 24% (95% CI 3–92%) and interlobular septal thickening in 23% (95% CI 1–80%) of the CT scans reviewed. Notable is the absence of cavitation. Similarly, another meta-analysis of 15 studies including 2451 patients did not report any cavitation, but commented on the development of traction bronchiectasis, consolidation, lymphadenopathy and pleural effusions at late stages of severe disease [7]. Reports from the previous two coronavirus epidemics, SARS-CoV and Middle East Respiratory Syndrome coronavirus (MERS-CoV), indicate similar patterns of radiological abnormalities with the development of bilateral, predominantly lower lobe, subpleural distribution of ground glass opacities and consolidation in both [8]. However, MERS-CoV had more severe inflammatory changes with reports of pleural effusions and only MERS-CoV reports noted the rare presence of pulmonary cavitation [9].

A small number published single patient case reports have more recently reported pulmonary cavitation [10–12], but this is considered a rare event. In contrast, experience at our tertiary care referral hospital differed. Therefore, we conducted a retrospective trial to study the incidence of pulmonary cavitation in patients with COVID-19, and to describe its characteristics and evolution.

## Methods

A registry of all patients admitted to our institution with COVID-19, and approved by Cleveland Clinic Abu Dhabi’s Research Ethics Committee, was retrospectively reviewed. All patients with COVID-19 admitted between February 23rd and July 3rd 2020 were identified. Baseline demographics, comorbidities, symptoms, ventilatory and other Intensive care parameters, microbiology, medications received and hospital outcomes were reviewed. Data was extracted by one of three members of the research team, and verified by a second researcher. All CT scans performed on these patients were identified and reviewed by a single thoracic radiologist noting imaging characteristics including the presence of pulmonary cavitation, the number, size, location/distribution of cavities, and features of fungal and other superadded infection.

Data are expressed as mean ± SD when normally distributed and as median and range when they are non-normally distributed. Normality of distribution was assessed using the Shapiro–Wilk test. Proportions were used as descriptive statistics for categorical variables. Statistical analysis was performed using Microsoft Excel 2019 for Windows software (with Real Statistics resource pack add-in).

In the absence of approved pharmacologic therapy for COVID-19, the Department of Infectious Diseases and the antimicrobial stewardship program at our institution rapidly developed institutional practice guidelines for COVID-19 based on available in-vitro and clinical studies to aid clinicians with treatment decisions. These guidelines were frequently updated based on emerging data. In the early stages of the pandemic asymptomatic and symptomatic patients were treated with a combination of two or three medications that included hydroxychloroquine, lopinavir-ritonavir and favipiravir, for a duration of 5–10 days. Tocilizumab was recommended for those with evolving cytokine release storm (CRS) based on criteria that encompassed clinical, radiological and laboratory parameters. One dose was initially given and a second dose was optional based on the clinical evolution of the patient. Empiric antibacterial and antifungal treatment was not routinely administered unless there was clinical suspicion for secondary pneumonia. Amoxicillin-clavulanate and piperacillin-tazobactam were the most common antibiotics used for COVID-19 patients. Systemic steroids when administered where as part of the ICU protocol for septic shock and not for treatment of COVID-19. All patients in our ICU with ARDS secondary to COVID-19 were ventilated following the ARDSNet protocol (low tidal ventilation mechanical ventilation strategy with 6 ml/kg of predicted body weight and a maintained plateau pressure below 30 cm H_2_O).

## Results

A total of 689 patients were admitted with COVID-19 pneumonia between February 23rd and July 3rd 2020, of whom 330 had asymptomatic or mild disease, 359 had evidence of pneumonia and 110 were admitted to Intensive Care for treatment of respiratory failure. One hundred and seventy eight patients had CT scans of the chest and all patients who had cavitary lung disease on CT scan were identified. Twelve patients with COVID-19 disease developed lung cavitation, comprising 1.7% (n = 12/689) of all admissions, 3.3% (n = 12/359) of patients who developed COVID-19 pneumonia, 7% (12/178) of patients who had CT scans, and 11% (n = 12/110) of patients admitted to the ICU. Table 1 describes their baseline characteristics, clinical variables and outcomes.

**Table 1:** Patient characteristics and outcomes of those who developed pulmonary cavitation

	Patient 1	Patient 2	Patient 3	Patient 4	Patient 5	Patient 6	Patient 7	Patient 8	Patient 9	Patient 10	Patient 11	Patient 12
Age range	50–59	40–49	60–69	40–49	60–69	50–59	40–49	30–39	40–49	50–59	30–39	40–49
BMI	30.9	30.4	31.3	29.2	25.1	29.4	26.3	21.5	30.1	19.8	24	27.7
Comorbidities	DM, HT	HT	DM	DM	COPD	HT	HT		DM, HT	DM	DM	DM
On admission												
Time from symptom onset to intubation (days)	6	12	9	7	5	5	8	15	3	9	10	8
P/F ratio	48.8	41.6	49.7	33.0	46.1	47.0	65.3	41.8	70.5	100.1	109.4	104.5
SOFA score (points)	24	21	24	25	23	23	16	21	25	23	18	17
Neutropenia											Y	
Leucocytes (× 10 * 9/L)	0.49	1.35	0.3	2.78	0.6	1.1	1.66	1.03	0.46	2.91	1.62	2.63
During the admission												
NM blockade	Y	Y	Y	Y	Y	Y	Y	Y	Y			Y
VTE		DVT and PE				PE	DVT			DVT		
CVA	Y									Y		
CRRT/IHD		Y	Y	Y	Y	Y			Y			Y
ECMO							Y	Y	Y			
Proned	Y	Y	Y	Y	Y	Y		Y	Y	Y		
Tracheostomy	Y		Y	Y	Y	Y	Y	Y	Y			
Days of systemic CS	17	15	19	24	11	14	14	25	18	9	6	5
+ ve fungal cultures or serology		Y	Y	Y		Y			Y			
Treated for fungal infection	Y	Y	Y	Y		Y	Y		Y			Y
Duration of hospital stay (days)	37	25	53	33	53	74	101	133	57	56	47	40
Outcome												
	Deceased	Deceased	Deceased	Deceased	Deceased	Deceased	Discharged home	Discharged home	Discharged home	Discharged home	Discharged home	Discharged home

*BMI* body mass index, *P/F ratio* PaO_2_/FiO_2_, *SOFA* sequential organ failure assessment, *NM* neuromuscular, *Y* yes, *VTE* venous thromboembolism, *DVT* deep vein thrombosis, *PE* pulmonary embolus, *CVA* cerebrovascular accident, *CRRT* continuous renal replacement therapy, *IHD* intermittent hemodialysis, *ECMO* extracorporeal membrane oxygenation, *CS* corticosteroids

Median (range) age was 47 (37–67) years, 50% (n = 6/12) had diabetes mellitus, 42% (n = 5/12) were hypertensive and one had chronic lung disease in the form of chronic obstructive pulmonary disease (COPD). None had received prior immunosuppression. All 12 were males, required invasive mechanical ventilation, and the median (range) time from symptom onset to invasive mechanical ventilation was 8 (3–15) days. Three were admitted directly via our institution’s Emergency Department and intubated within 24 h, and nine patients were transferred from other hospitals within 72 h of intubation. At the time of this data review six of the 12 patients had died, and six discharged home.

Most patients had completed a course of Hydroxychloroquine (83% n = 10/12) and antivirals (favipilavir or lopinavir/ritonavir) (92%, n = 11/12) in the early stages of their illness. All 12 patients received Tocilizumab as clinical evidence of a CRS became apparent accompanied by deteriorating respiratory status and progressively worsening disease on imaging. Overall, this represented 9% (n = 12/133) of patients with COVID-19 who received tocilizumab in our institution.

### Imaging characteristics

Upon admission all patients had baseline chest X-rays which did not reveal any evidence of cavitary disease. All 12 patients had the recognized range of imaging features expected in severe COVID-19 lung disease with; bilateral ground glass opacities predominantly in the peripheries, some with centrally located opacities, most had consolidation and air bronchograms, and roughly half had crazy paving pattern and interlobular septal thickening. In 10 patients all five lobes were affected, and in two patients the disease spared two lobes. The median (range) number of days between symptom onset and the first CT demonstrating cavitation was 36 (21–54) days, and between intubation and the first CT demonstrating cavitation 28 (13–49) days. Table 2 describes the cavities in detail. In short, five of the 12 patients had solitary cavities with size ranging between 30 and 100 mm in diameter. All patients with more than one cavity had bilateral cavitation. The appearances and morphology of the cavities amongst the group were similar with to that of pulmonary abscesses with thick but smooth walls containing internal debris and air fluid levels. Pulmonary infarcts were excluded as the CT studies were performed with IV contrast excluding pulmonary emboli. All five lobes contained cavities in a similar proportion, with a predilection for the costophrenic angles and the apices.

**Table 2:** Characteristics of the pulmonary cavities and related events

	Patient 1	Patient 2	Patient 3	Patient 4	Patient 5	Patient 6	Patient 7	Patient 8	Patient 9	Patient 10	Patient 11	Patient 12
No. of cavities (n)	1	8	8	3	1	1	2	1	9	3	1	5
Largest cavity (mm)	50	85	60	30	30	30	70	100	54	52	50	40
Bilateral		Y	Y	Y			Y		Y	Y		Y
Location of cavities												
No. lobes with cavities (n)	1	5	5	3	1	4	2	1	4	3	1	3
RUL		Y	Y	Y				Y	Y	Y	Y	
RML		Y	Y		Y	Y			Y			
RLL		Y	Y			Y	Y		Y	Y		Y
LUL		Y	Y	Y		Y	Y		Y			Y
LLL	Y	Y	Y	Y		Y				Y		Y
Clinical events												
Developed pneumothorax				Y				Y	Y			Y
Developed hemoptysis			Y				Y		Y			Y
Treated for Invasive fungal infection	Y	Y	Y	Y		Y	Y		Y			Y
Bacterial organisms in Sputum/BAL	*K. pneumoniae*	*ECC*	*MRSA*		*S. maltophilia, C. koseri*	*MSSA, S. maltophilia*	*S. marcescens, S. maltophilia, ECC*	*K. pneumoniae*	*Acinetobacter, MRSA*	*ESBL K. pneumoniae, MRSA*	*MRSA*	*K. pneumoniae*

*Y* yes, *RUL* right upper lobe, *RML* right middle lobe, *RLL* right lower lobe, *LUL* left upper lobe, *LLL* left lower lobe, *BAL* bronchoalveolar lavage, *MRSA* methicillin-resistant *Staphylococcus aureus*, *MSSA* methicillin-sensitive *Staphylococcus aureus*, *K. pneumoniae* Klebsiella pneumonia, *ECC* enterobacter cloacae complex, *S. maltophilia* *Stenotrophomonas maltophilia*, *S. marcescens* *Serratia marcescens*, *ESBL* extended spectrum beta-lactamase

## Discussion

The development of pulmonary cavitation in patients with severe COVID-19 lung disease treated in our institution’s ICU was not a rare event (11%, n = 12/110). This subgroup of patients had very severe infection with acute respiratory distress syndrome (ARDS) and required a prolonged ICU stay. Median (range) Sequential Organ Failure Assessment (SOFA) score was 23 (range 16–24) on admission, and all patients were leucopenic. Seven of 12 required renal replacement therapy, four developed venous thromboembolism, three required ECMO with all surviving to successful decannulation, and two had thromboembolic cerebrovascular events.

**Fig. 1:**
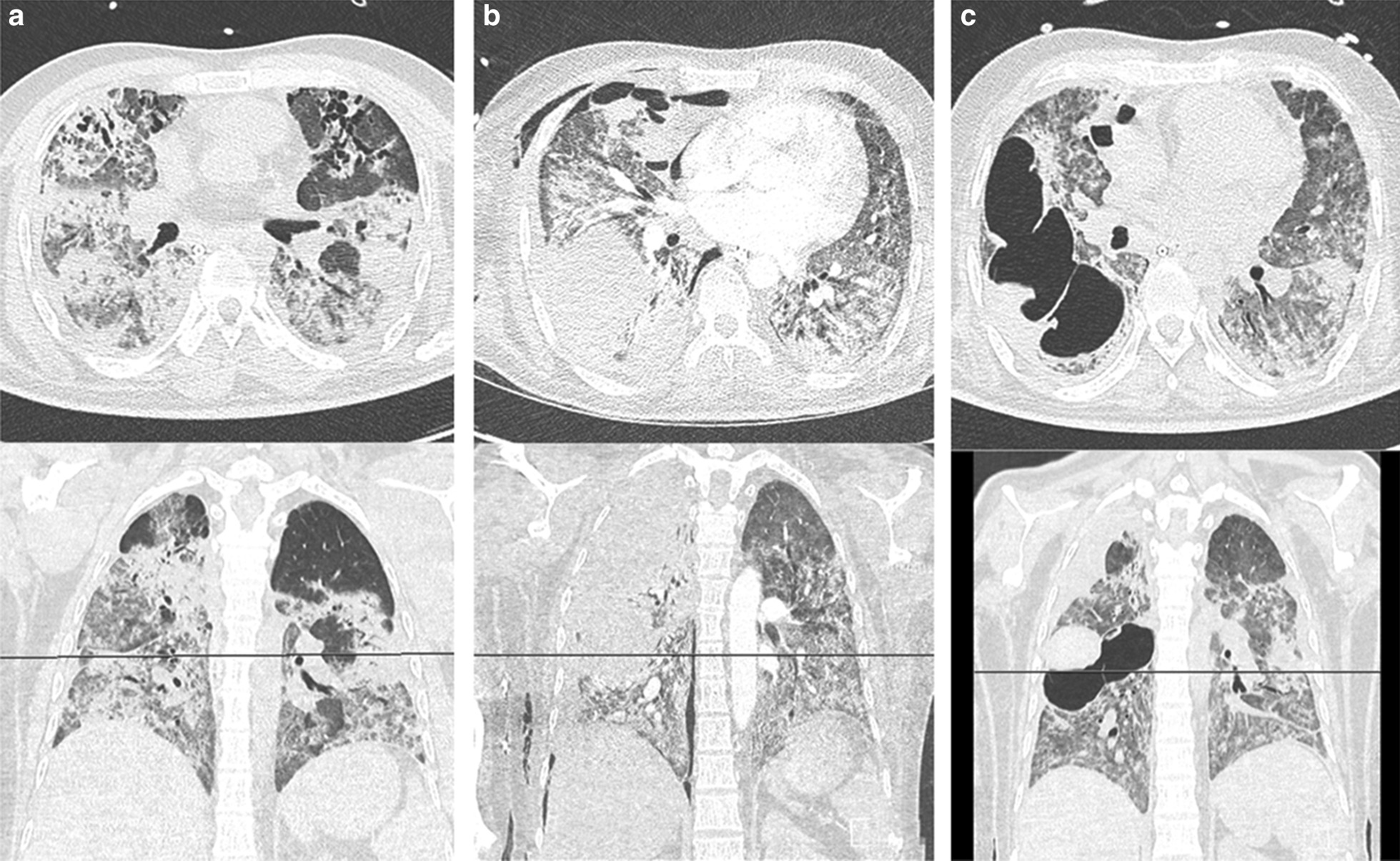
Axial and coronal CT images at form patient 8 on **a** 5th May 2020, **b** 26th May 2020, and **c** 6th June 2020

**Fig. 2:**
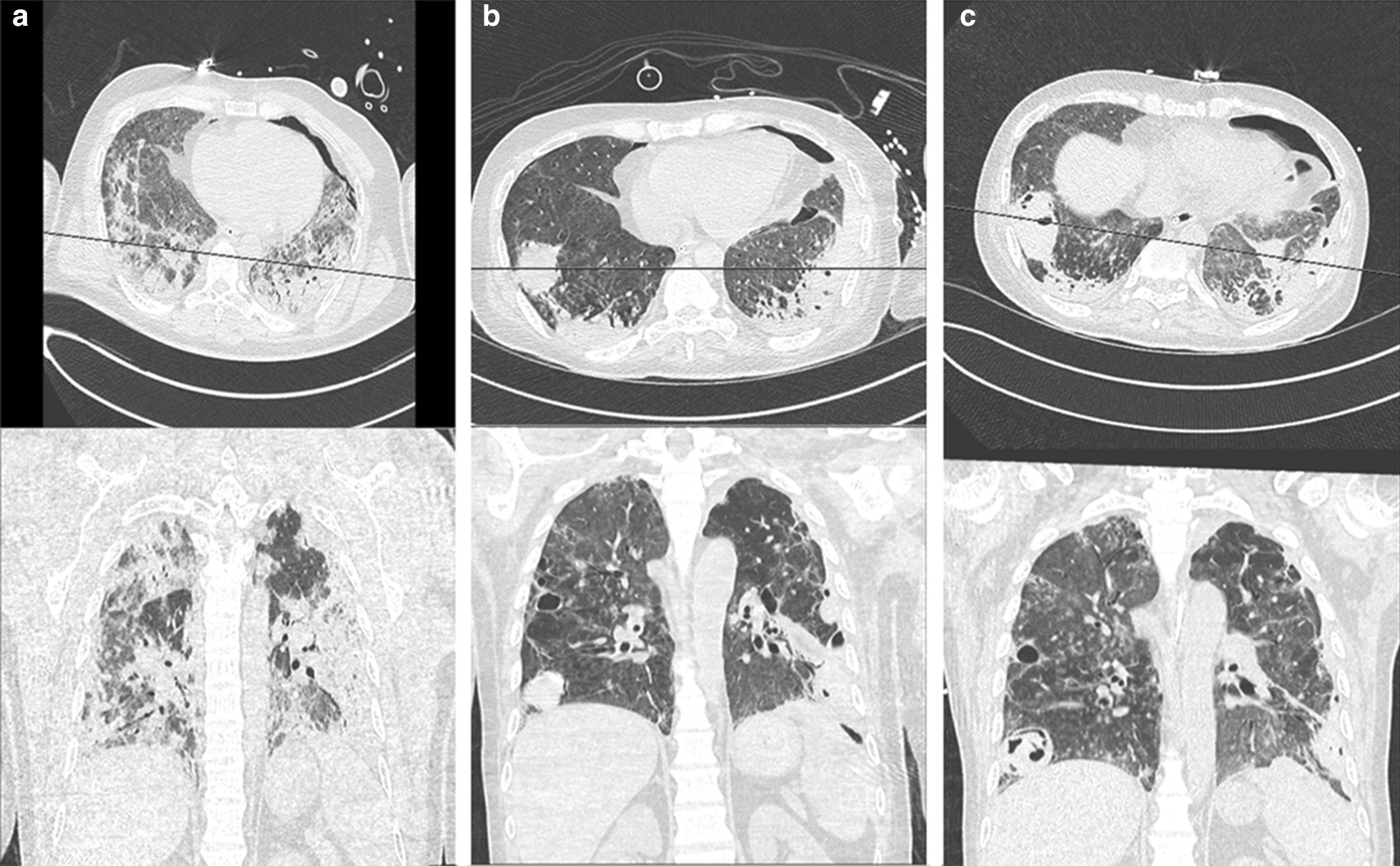
Axial and coronal CT images at form patient 9 on **a** 14th May 2020, **b** 8th June 2020, and **c** 18th June 2020

By definition, a cavity is an air-filled space forming within an area of pulmonary consolidation, mass or nodule, as a result of liquefication of the necrotic portion of the lesion and the discharge of this necrotic material via the bronchial tree. This exact process occurred in our patients as cavities formed in areas of the lung where ground glass opacities were seen in early stages, morphing into more dense consolidation, later developing necrosis and ultimately cavitating. This is demonstrated for patients 8 and 9 in Figs. 1 and 2, respectively. It is uncommon for viral pneumonias [13], including those due to the other human coronaviruses SARS-CoV [14] and MERS-CoV [15], to cause pulmonary cavitation even in severe and advanced viral infection. We are unable to speculate as to whether bacterial infection and/or invasive fungal coinfection may have contributed to the development of the cavities, or if the infections were opportunistic. Four of twelve patients who had developed pulmonary cavitation (including two of the survivors) had no microbiological, serological, clinical or distinct radiological characteristics of invasive fungal infection and did not receive treatment for this. However, these four patients did have infection with bacterial organisms known to cause cavitation. Infection with mycobacterium tuberculosis (MTB) is also a common cause of lung cavitation and in a recently published case series [16], it has been described as a coinfection in COVID-19 patients resulting in cavity formation. However, in all 12 of our patients, MTB infection was ruled out based on negative Acid-Fast Bacilli on smear and culture of multiple respiratory specimens.

All patients in this series received tocilizumab, a recombinant humanized monoclonal antibody directed against both the soluble and membrane-bound forms of the interleukin-6 (IL-6) receptor, in the early stages of a CRS. Tocilizumab is currently approved by the US Food and Drug Administration (FDA) for the treatment of severe rheumatoid arthritis, systemic juvenile idiopathic arthritis, giant cell arteritis, and life-threatening CRS induced by chimeric antigen receptor T cell therapy [17]. Recently it has been associated with improved survival in patients with severe COVID-19 pneumonia with evidence of CRS [18, 19]. In general tocilizumab is well tolerated but can induce neutropenia, and an increased risk of developing infections has been reported [20, 21]. Furthermore, it may predispose to a delay in detecting active infection because of the masking effect of a suppressed C reactive protein (CRP) response. Interestingly, however, only one of 12 patients in our cohort developed neutropenia during their ICU stay. All patients in this series received systemic glucocorticoids, which may have survival benefit in COVID-19 [22, 23], but also suppress the immune system by impairing innate immunity. In the treatment of patients reported here systemic steroids were administered as part of our ICU protocol for septic shock, based on the Society of Critical Care Medicine guidelines [24] and not directly for the treatment of their COVID-19.

We therefore hypothesize that the causes of cavitation in these patients was multifactorial, with contributing factors including: bacterial and fungal co-infection; the immunosuppressive effects of glucocorticoids and tocilizumab; SARS-CoV-2 specific inflammatory pathways; the COVID-19 related predisposition to venous thromboembolism and potential to cause infarct and micro-infarcts leading to cavitation; and the severe morbidity of this patient population.

Four patients developed hemoptysis and all had features of suspected invasive aspergillosis. Hemoptysis appeared to have occurred irrespective of cavity size. Similarly, secondary pneumothorax also occurred in patients with both larger and smaller cavities.

The high level of morbidity and mortality in this small case series highlights that cavity formation probably sits at the severe/end-stage of the radiological COVID-19 spectrum. It is unclear what the natural history of these cavities will be in survivors. This will be informed by future follow-up interval imaging but it is reasonable to assume that though there may be some regression in the size of the cavities, there will be an increased future risk of pneumothorax, hemoptysis, colonization with bacteria including non-tuberculous mycobacteria, fungi and the development of mycetomas. The development of pneumothorax has been reported but is uncommon in COVID-19 patients. Shan et al. reported [25] a case of a patient who developed pneumomediastinum, pneumothorax and subcutaneous emphysema, while Sahu et al. [26] described a patient with COVID-19 infection who developed pneumopericardium. Neither of these two cases had cavitary lung disease, and hence alveolar damage was the likely cause of the development of pneumothorax and pneumopericardium. In our cohort of patients with cavitary lung disease, four out of 12 developed pneumothoraces. Therefore it is reasonable to conclude that cavitary lung disease will increase the risk of pneumothorax, likely by extension of cavitary lesions to the pleural surface, by rupture of the thin cavity walls as a result of fibrosis and scarring of the lung and resultant remodeling and tethering, especially when pleural adhesions exist.

Limitations of this study include its retrospective observational nature and the changing patient population in terms of severity of disease being admitted to hospital (and hence included in the registry) over time. The decision to obtain cross sectional imaging was clinically guided and not protocolized, and hence the study may have underestimated the true prevalence of pulmonary cavitation if some had formed in patients who did not have CT scans. Finally, treatment protocols changed over time as novel clinical evidence became available as the pandemic evolved, and the use of antivirals, hydroxychloroquine, corticosteroids, and the treatment of superadded infections was not standardized.

## Conclusion

This study highlights that pulmonary cavitation in patients with severe COVID-19 lung disease can occur, is associated with secondary complications of hemoptysis, pneumothorax, and confers a poor prognosis. Early cross sectional imaging should be considered if there is suspicion of cavitation on plain radiographs, and a more aggressive investigation and treatment of possible invasive fungal infection undertaken. Further studies are needed to determine whether treatment with tocilizumab, systemic glucocorticoids or a combination of both may increase the risk of developing pulmonary cavitation in patients with COVID-19.

## Data Availability

The datasets used and/or analysed during the current study are available from the corresponding author on reasonable request.

## Abbreviations

ARDS: Acute respiratory distress syndrome
COPD: Chronic obstructive pulmonary disease
COVID-19: Corona virus disease 2019
CRP: C-reactive protein
CRS: Cytokine release storm
CT: Computerized tomography
ECMO: Extra-corporeal membrane oxygenation
FDA: Food and Drug Administration
ICU: Intensive care unit
IL-6: Interleukin-6
MERS-CoV: Middle East Respiratory Syndrome coronavirus
MTB: Mycobacterium tuberculosis
SARS-CoV-2: Severe acute respiratory syndrome coronavirus 2
SOFA: Sequential Organ Failure Assessment

## References

[1] World Health Organization. Coronavirus disease 2019 (COVID-19) situation report—156. https://www.who.int/docs/default-source/coronaviruse/situation-reports/20200624-covid-19-sitrep-156.pdf?sfvrsn=af42e480_2.

[2] Song F, Shi N, Shan F (2020). Emerging 2019 novel coronavirus (2019-nCoV) pneumonia. Radiology.

[3] Kong W, Agarwal PP (2020). Chest imaging appearance of COVID-19 infection. Radiol Cardiothorac Imaging.

[4] Li X, Zeng X, Liu B, Yu Y (2020). COVID-19 infection presenting with CT halo sign. Radiol Cardiothorac Imaging.

[5] Salehi S, Abedi A, Balakrishnan S, Gholamrezanezhad A (2020). Coronavirus disease 2019 (COVID-19): a systematic review of imaging findings in 919 patients. Am J Roentgenol.

[6] Zheng Y, Wang L, Ben S (2020). Meta-analysis of chest CT features of patients with COVID-19 pneumonia. J Med Virol.

[7] Sun Z, Zhang N, Li Y, Xu X (2020). A systematic review of chest imaging findings in COVID-19. Quant Imaging Med Surg.

[8] Chen X, Zhang G, Hao S, Bai L, Lu J (2020). Similarities and differences of early pulmonary CT features of pneumonia caused by SARS-CoV-2, SARS-CoV and MERS-CoV: comparison based on a systemic review. Chin Med Sci J.

[9] Das KM, Lee EY, Langer RD, Larsson SG (2016). Middle east respiratory syndrome coronavirus: what does a radiologist need to know?. Am J Roentgenol.

[10] Amaral LTW, Beraldo GL, Brito VM (2020). Lung cavitation in COVID-19: co-infection complication or rare evolution?. Einstein Sao Paulo Braz.

[11] Selvaraj V, Dapaah-Afriyie K (2020). Lung cavitation due to COVID-19 pneumonia. BMJ Case Rep.

[12] Xu Z, Pan A, Zhou H (2020). Rare CT feature in a COVID-19 patient: cavitation. Diagn Interv Radiol Ank Turk.

[13] Koo HJ, Lim S, Choe J, Choi S-H, Sung H, Do K-H (2018). Radiographic and CT features of viral pneumonia. RadioGraphics.

[14] Wong KT, Antonio GE, Hui DSC (2003). Severe acute respiratory syndrome: radiographic appearances and pattern of progression in 138 patients. Radiology.

[15] Ajlan AM, Ahyad RA, Jamjoom LG, Alharthy A, Madani TA (2014). Middle East respiratory syndrome coronavirus (MERS-CoV) infection: chest CT findings. AJR Am J Roentgenol.

[16] Yousaf Z, Khan AA, Chaudhary HA (2020). Cavitary pulmonary tuberculosis with COVID-19 coinfection. IDCases.

[17] Tocilizumab (Actemra) injection, for intravenous or subcutaneous use (Genentech Inc, San Francisco, CA). Prescribing Information. https://www.gene.com/download/pdf/actemra_prescribing.pdf. Accessed 6 July 2020.

[18] Toniati P, Piva S, Cattalini M (2020). Tocilizumab for the treatment of severe COVID-19 pneumonia with hyperinflammatory syndrome and acute respiratory failure: a single center study of 100 patients in Brescia, Italy. Autoimmun Rev.

[19] Guaraldi G, Meschiari M, Cozzi-Lepri A (2020). Tocilizumab in patients with severe COVID-19: a retrospective cohort study. Lancet Rheumatol.

[20] Pawar A, Desai RJ, Solomon DH (2019). Risk of serious infections in tocilizumab versus other biologic drugs in patients with rheumatoid arthritis: a multidatabase cohort study. Ann Rheum Dis.

[21] Feist E, Burmester G-R (2009). Is tocilizumab in combination with traditional DMARDs safe and effective for patients with active RA?. Nat Clin Pract Rheumatol.

[22] Horby P, Lim WS, Emberson J (2020). Effect of dexamethasone in hospitalized patients with COVID-19: preliminary report. medRxiv.

[23] Beigel JH, Tomashek KM, Dodd LE, Mehta AK, Zingman BS, Kalil AC, Hohmann E, Chu HY, Luetkemeyer A, Kline S, de Castilla DL (2020). Dexamethasone in Hospitalized Patients with Covid-19—preliminary report. N Engl J Med.

[24] Rhodes A, Evans LE, Alhazzani W (2017). Surviving sepsis campaign: international guidelines for management of sepsis and septic shock: 2016. Crit Care Med.

[25] Shan S, Guangming L, Wei L (2020). Spontaneous pneumomediastinum, pneumothorax and subcutaneous emphysema in COVID-19: case report and literature review. Rev Inst Med Trop São Paulo.

[26] Sahu KK, Mishra AK, Goldman Y (2020). A rare case of pneumopericardium secondary to COVID-19. Heart Lung J Cardiopulm Acute Care.

